# Observations on Cholera in Military Practice

**Published:** 1852-08

**Authors:** W. H. Tingley

**Affiliations:** Late an officer of the Medical Staff, U. S. Army, Member of the Academy of Natural Sciences, etc.


*Observations on Cholera in Military Practice.* By W. H. Tingley, M. D., late an officer of the medical staff, U. S. Army, Member of the Academy of Natural Sciences, etc.

Of the many diseases that have claimed the attention of the medical profession, few have found so prominent a place as Epidemic Cholera. It has existed under circumstances, to all human knowledge, the most dissimilar, and exhibited an irregularity of developement and progress that has alike defied the skill of medicine, and the researches of pathology. Many hypotheses have been constructed to explain its various phenomena, but few have approximated probability, fewer still have had a semblance of truth.

There are causes operating which are often coincident, yet do not produce disease; these obtain throughout the imponderable world, effecting daily and hourly changes in the vital forces; they are too subtle to be appreciated—too mysterious for human view, and into which no eye shall see, and of which no tongue shall speak. Yet if we cannot grasp the substance we may seize the shadow, and learn in time by patient observation what at first seemed insurmountable. Reason may prove fallacious, but well digested facts are 'built upon a rock.'

If the observations made by the writer, under trying and peculiar circumstances, can add aught to the already accumulated stock, he will rest satisfied that he has not labored in vain.

The command to which I was attached, consisting of three hundred and twenty men, sailed from Fort Wood, New York Harbor, April 2d, 1851, in a well and comfortably fitted transport. We arrived at New Orleans Barracks after a tedious voyage of twenty eight days, without any important sickness. From this place we embarked in a steamer for Jefferson Barracks, on the Mississippi, ten miles below Saint Louis, where we arrived in eight days, in good health. Thence we sailed in a small, dirty, and crowded steamer for Fort Leavenworth in the Indian Territory, on the Missouri, five hundred miles above its confluence with the Mississippi. On board the steamer there were many returning Californians, whose total disregard for personal cleanliness was remarkable. Four days after our departure the weather which had been mild and pleasant, became sultry, wet and warm. At this period diarrhœa appeared among the men, and two days afterwards three were attacked with unequivocal malignant cholera; on the following day two more men were taken down; of all these, two died, one in five, and the other in eight hours; these two men were the most intemperate and insurbordinate in the command. On our arrival at Fort Leavenworth the subsequent day, the command was marched to camp 'Burgwin,' three miles west of the Fort, where two hundred and fifty men had arrived, a fortnight before, from Newport Barracks, Kentucky. They had passed down the Ohio and up the Mississippi and Missouri rivers, but had no sickness among them, nor had they any at the time of our arrival, excepting the ordinary camp diarrhœa which attacks most soldiers when first taking the field. On the day after our arrival the cholera broke out among these men, and from this time it became rife. The site of the camp was in the basin of an undulating prairie, surrounded by alternately receding acclivities, and deeply furrowed water courses, through which the rain streamed during the heavy showers, which now fell almost hourly, into the plain below, forming pools of water in and around the company's tents and drill ground. The soil is a rich loam, based on a substratum of argillacious mould, and limestone. Several species of Nymphaceæ grow in the ponds and the Lycopodaceæ on the hill sides. The principal trees are *Juglans nigra*, *Carya olivæformis*, *Cayra alba*, *Acer saccharinum*, *Plantanus accidentalis*, *Cerasus virginianæ*, *Morus rubra*, *Quercus alba*, and *Populus canadensis*. A luxuriant growth of grass covers the lowlands, and the Silphium lancinatum (Pilot weed) abounds. The prevailing winds were from south and southeast. The average temperature, for one week was (in round numbers) 72° Fahr. during the day, and 56° during the night. The greatest daily extremes were 83° and 58°. The greatest nightly extremes, 56° and 50°. The weekly average of Hygrometric observations, showed the 'dew point' at 66°, its greatest extremes 70° and 63°. The hygrometic observations were made at daylight, and 3 P. M., (according to Kœmptz, the relative humidity of the atmosphere is greatest at daylight.) The weekly average of the barometer was 29.231, its greatest extremes 29.854 and 28.105.

The tension of the atmospheric electricity was considerably diminished; this condition is nearly always coincident with a high 'dew point.' The electricity of the atmosphere, like the barometer, undergoes several *horary* variations. According to Becquerel, its least intensity is at daylight, and 3 P. M. At these respective hours the majority of cholera cases were reported, more, however, at the former than the latter hour. The sound of the reveille drum frequently brought the hospital steward to my tent to say, 'another man just taken down.' The disease began to decline as soon as the weather became settled—when the relative humidity of the air was diminished, and when we had crossed the valley of the Kansas river, and began the ascent to the upland 'plains.'

According to Cavallo, the atmospheric electricity is weakest in hot weather, and previous to a shower of rain. The observations of Mr. Howard have proved—first, that the positive electricity common to fair weather disappears and yields to a negative state before rain. Secondly, that the rain that falls first after a depression of the barometer is negative. To these, I can add my own observations. The night on which the disease was most rife, was the most terrific I have ever witnessed. The rain fell in torrents, swelling the stream near our camp, which we had crossed nearly dry shod a few hours previous, to its highest banks. The fitful blasts howled through the thick foliage of the forests trees, overturning in its resistless course many tents, and exposing their inmates to its merciless rage. The lightning played with incessant glare, and the thunder roaring far and near, mingled mournfully harsh with the shrieks and agonising cries of the dying. On that night the spirit of pestilence and the spirit of the storm stalked forth arm in arm.

Dr. Crawford,(Observations on the Asiatic Cholera in St. Petersburg,) says: "During the latter end of May, and the whole of June, a remarkable change took place in the weather. There were almost constant high winds, shifting frequently and suddenly round to every point of the compass, and often accompanied by torrents of rain, and sometimes thunder. This disturbed state of the atmosphere was indicated by sudden fallings and risings of the barometer, sometimes to the extent of between one and two inches. The changes of the temperature were equally frequent and rapid, the heat being for several days very great, as high as 84° to 90° Fahr., and the air exceedingly sultry and oppressive, and a damp, relaxing south wind; and then suddenly on a change of wind, and sometimes on the occurrence of a thunder storm, this oppressive heat would be succeeded by great cold, the thermometer falling as much as 50° in a few hours, so that it was in June several times below the freezing point. Another peculiarity in the condition of the air, was the disturbed state of its electricity. This was clearly demonstrated by the fact, that the electric machines could not be charged, and to a great extent lost their power, which generally happens whenever the atmosphere is damp and unsettled." Muller takes a somewhat opposite view. (*Einige Bemerkungen über die Asia-*

*tische Cholera.*) He says, "Throughout the whole duration of the epidemic, the air was oppressive, changeable, and heavy. Thunder storms occurred often, but had no influence on the number of attacks or recoveries. Rain fell almost daily, the heavens were gloomy, the evenings foggy, and the sun was seldom visible." Dr. Muller does not say whether he kept a daily record of the state of the atmospheric electricity. When in China, several years since, I was informed by an officer of the British army, that before and during the malignant fever which ravaged the island of Hong Kong the year previous to my visit, 1843, the weather was variable, alternately hot and cold with heavy rains, and that there was no 'electricity in the air.'

I have been informed by my friend Lieutenant Sturgis, 1st. U. S. Dragoons, that a detachment of men under his command which started from New York in May, 1850, for Fort Leavenworth, via the Lakes, had no sickness until they arrived at Lasalle, Illinois, after they had been for several days crowded in a small and dirty canal boat. On their arrival at the aforesaid place, the cholera broke out among them and continued with the command, until they had crossed the valley of the Kansas river. Although there was no meteorological register kept, Lieut. S. says, 'the weather was hot and rainy during the day, and cold at night.'

The principal conditions under which the electricity of the air is developed, require but a passing notice. They are friction of the air against the earth. Change of state in fluids—evaporation; it is evolved during the germination of plants, and the breaking of crystals.

My experience strongly inclines me to seek for the causes of cholera, and many, if not all, diseases of a pestilential nature, in the irregularities and diminished tension of the atmospheric electricity. The agency of these changes in producing a predisposition to disease may be considered in a three fold relation. First, as acting, *per se*, separate and apart from all correlative conditions. Second, as influencing the condition of the efficient causes, whether vegetable, animal, geological, or fungous in their nature, and predisposing the body to the operation of one or more of these. Third, as acting in combination with one more of the above. In the barren and uncertain state of statistical knowledge of the effects of physical agents on the animal constitution, the difficulty of selecting one of these conditions above the rest will be apparent. The first proposition may with propriety be rejected, because the laws of nature are immutable, and when determined we have a *point depart* for judging of analogous states.

The laws which regulate our sphere, and the thousands of worlds surrounding it, have been in operation since 'time was.'

'Such as creation's dawn beheld
Behold thee now.'

One drop of water less in the world than when it was created, would cause an eccentricity in its motion, whose consequences might prove a 'wreck of matter.'

The compass that guides the mariner, has its own fixed principles. Light has its own laws. If these were changing, excepting according to known and regular progression, the interests of commerce, of navigation, and of science would be forever destroyed. The barometer in its mutations is faithful and constant, predicting to a certainty the coming tempest, and enabling the mariner to protect his ship and his life against the 'strife of elemental war.' Why should not electricity, in its relation to organized matter, have its fixed laws likewise? No one can doubt that the identical state of the atmospheric electricity which at one time produces pestilence, at another time may be compatible with the most vigorous health. The choice therefore lies, necessarily, between the second and third propositions. Here the difficulty is increased tenfold. I shall, however, select the second, because under the circumstances of the first appearance of the disease among our men, there was sufficient cause for the developement of a pestilence that had for several days foreshadowed its invasion. If we assume that the active cause was of a vegetable character, we certainly had enough decaying vegetable matter and filth to prove a *foyer* of disease. If it is admitted that it is animal in its nature, there is a ready explanation in the crowding together of so many men in a narrow and imperfectly ventilated space, and whose personal cleanliness was totally disregarded. If the fungous theory is viewed as causing a proclivity to pestilential disease, we might have found conditions amply efficient. Furthermore, opposed to the last proposition, which presupposes immediate action under concurrent conditions of air and earth, is the fact that the men complained of cramps in the upper and lower extremities, of lassitude, debility and vertigo, which is sufficient to prove the action of this subtle agent on the human frame. Dr. Holland, (Medical Notes) says: "One of the best tests of the actual operation of the atmospheric electricity on the body is, as I think, that mixed sensation of heat and cold which most persons must recollect at some time to have felt—or rather the consciousness of sensations which cannot clearly be defined to be either. Concurrently with such a state of atmosphere which the thermometer does not in any way interpret to us, there generally occurs more or less of the lassitude before described, the muscles are readily fatigued, some degree of headache is often felt, and other vague uneasiness of the bodily feelings, varying much in different habits, and doubtless influenced by the condition of the health at the time."

I believe that the subtle agent (electricity) primarily affects the nervous system through the medium of the lungs, and that the blood disorder is a consequence of irregular innervation. We see this in the diminished power of calorification, and the depressed and disordered circulation. It is under such conditions that efficient causes may require a force and intensity that under a positively electrified state of atmosphere they do not possess. The *prodromes* were cramps, lassitude, debility and vertigo; the biliary secretion was suppressed, and the portal circulation congested, as evidenced in the clay colored stools, discharges of blood from hemorrhoidal tumors, bitter pasty taste in the mouth, as if the patient had 'eaten a bad egg,' or 'drunk bilge water,' alternately constipation and diarrhœa. If we admit that the impression of the morbific agent is made on the nervous system, and, following this, on the blood, we can readily account for the hæmatological changes and the irregular transmission of nervous power, so well marked in cholera.

Those who have witnessed the enormous discharges from the intestinal mucous membrane, and the suddenness with which the cholera often makes its invasion, will not doubt a two fold operation of the efficient cause or causes. To those who doubt it, and view the nervous and muscular symptoms as sequacions to primary action in the blood, I would only ask, if these should not show marked effects only after changes noticed to the blood—should not blood drawn from a vein during the *Prodromes*, show some physical or chemical change. The former I have never observed; the latter I had not the means of observing.

Prevost found that an electric spark passed through a drop of blood, caused the particles it contained to partially separate from their elementary globules.

The speedy course of the disease is sometimes remarkable. Twice I have seen cavalry soldiers, apparently in good health, fall from their horses and die within an hour. Two men who died thus suddenly, I know were and had been in good health for ten or twelve days. With the exception of hydrocyanic acid there is no agent, save electricity, that can so entirely disorganize the blood, and this agent must first produce its effect on the great nervous centres.

The question of the propagation of cholera by contagion has claimed no inconsiderable attention. Many singular facts have been adduced by the contagionists and non-contagionists. Surgeon Thomas Lawson, now Surgeon General, in his official report, 1832, says: "No case of the disease manifested itself at New Orleans until the arrival in port of the steamer 'Constitution,' which had several cases on board—a number of the passengers having already fallen victims to the disease; so fearfully rapid was the pestilence in its progress, than in less than forty-eight hours it reached the lowest plantation on the Mississippi, desolating almost every spot inhabited by man. Like a skilful general, it seems to have advanced upon the capital, leaving the minor posts on the line of march untouched. Whatever may have been the cause of the disease, it was unknown among us until the steamer Constitution arrived in port."

There had been no cholera in any of the towns on the Mississippi river, and it was not until several days after our arrival at Fort Leavenworth, that it appeared in Independence, Missouri. Nor had there been any cases at the Fort. For two months the disease lingered about the river towns, and nearly every steamer passing up and down the river had more or less of the pestilence on board. On our march we overtook a train of Santa Fe traders who, up to this time, had been in excellent health; after this the disease broke out among them, but not with great malignity.

Many of the hospital orderlies were attacked while attending the sick. At a Pottawattomie Indian settlement near which we passed, the disease raged for a week with fearful mortality. Assistant Surgeon Kennedy and myself were attacked nearly at the same time, without any premonition, while attending to our professional duties. He died in six hours; I suffered a long and tedious convalescence. Company B., First U. S. Dragoons, passed over our route a fortnight after us, and had the same inclement weather, yet not one man was reported sick; but they were old and acclimated soldiers, well used to the field. From these and similar facts in the practice of other medical officers, I was induced to believe that cholera is generally *contagious*, but that under favorable circumstances it may be *non-contagious.*

In treating this disease two methods were employed—the sedative, and the stimulant and alterative. The first consisting of opium and acetate of lead, was adopted by my colleague, Assistant Surgeon Kennedy. The last method was followed by myself. The combination was calomel in large proportions, (I have found this drug in large doses, xx. grs., relieve the nausea and irritability of stomach), camphor, capsicum and quinine, and small quantities of acetate of morphia. In a few cases, I gave calomel combined with rhubarb and ipecacuanha; in civil practice this treatment may be beneficial in some cases, but soldiers and sailors require more heroic medication. Those cases which terminated fatally, treated by the first method, usually died during the stage of collapse; those who died after the treatment by the second method died either during the stage of reaction, or from some intercurrent or consecutive disease, as apoplexy, typhoid fever, pneumonia, or gastro-enteritis. When I saw a patient before the *algide* state commenced, the comparative ratio of deaths was as 42 to 54. Those who have not witnessed the destitute and comfortless condition of troops on a march, cannot readily understand how mortality can be so great during an epidemic. Within a few days, I have heard from a detachment of troops which left in May for Santa Fe. They had had the cholera, and the mortality was one out of every eight men composing the command. I believe this treatment would be more successful in private practice, where such valuable adjuvants as warm and vapor baths can be obtained, to say nothing of the advantage of houses over tents for invalids. After a trial of several methods of treatment, I am satisfied with the superiority of the stimulant and alterative treatment above detailed. From what I have seen of the opium treatment, I am not disposed to advocate it. When it is beneficial it is in the very early stages of the disease, when there has been precursory diarrhœa, and before the characteristic rice water discharges appear. I have seen the error of 'locking up the bowels,' and retaining the morbid secretions of the intestinal canal in contact with its mucous membrane. Moreover, I have seen patients *overdrugged* more than once by not very large doses of this medicine. The opium treatment is emphatically empirical—it combats symptoms, not the disease. When the nature of the disease is changed by calomel, quinine, &c., or by a "crisis'—as soon as the alvine evacuations become fœcal and bilious, the mineral astringents (acetate of lead I prefer) exert a salutary and tonic influence on the gastro-intestinal membrane. I have tried several of the vegetable astringents, but have no faith in them.

In a few cases I bled, not empirically, but to relieve cerebral congestion—this was never urgent save in the reactive stage, when the injected conjunctiva, pain across the temples, heaviness of intellect, and partial coma, indicated the condition of the cerebral circulation. Spasmodic action of the muscles, and cramp were relieved by sinapisms, and towels wrung out of hot vinegar. I have tried chloroform, and have the fullest reasons for being dissatisfied with it. Sometimes cholera assumes anomalous forms. An officer of the staff was attacked and went through the disease without having more than five or six evacuations per *anum*; he however, ejected per *orem*, matter in all respects resembling 'black vomit,' and was so near death that he had involuntary alvine discharges, a state usually considered beyond hope. He suffered severely for several weeks with gastralgia, cardialgia, and a paralytic condition of the lower portion of the colon. I prescribed friction with dry mustard to the abdomen, diluted tincture of cantharides to the spinal column, small doses of brandy and the alcoholic extract of nux vomica, until urethral irritation was induced; under this treatment he gradually recovered.

I made but few necroscopic examinations, and those, made after the fatigues of a march, were necessarily hasty. In those who died during the stage of collapse, the mucous membrane of the stomach and intestinal canal was pale, soft, and easily detached from the subjacent tissue. The liver was anæmic, and the gall bladder filled with thick bile. In those who died during the stage of reaction, there were traces of *diptheritic* inflammation, enlargement of the mucous follicles, and engorgment of the mesenteric veins. In one case there was found a state of red hepatization of the lungs; this condition was suspected during life. My attention was first directed to it by the comatose condition of the patient, which evidently did not arise from the disease, (cholera). The engorged upper lobes pressed upon and obstructed the blood in the superior vena cava.

There has not been much written respecting the *sequelæ* of cholera; some have considered abdominal typhus as one; others a well developed *gastro-enteritis* as another. My own observations do not coincide with either of these views. I have never seen the tenderness about the right iliac region which marks the first, nor the pain and tenderness about the umbilicus, so characteristic of the other; but I have seen an accelerated pulse, dry, harsh skin, furred tongue, morbidly increased appetite and diarrhœa, which are prominent symptoms of a low form of intestinal inflammation; and this condition I consider the true consecutive disease. In a few cases the affection of the duodenum extends to the biliary ducts, and to the stomach, but the gastric disorder is strictly functional, and consists in disordered secretion of the gastri follicles, and atony of the muscular coat. The disordered stomach is soon relieved, but the intestinal disease is present even after many years. The brain is more or less affected—dimness of vision, vertigo, and mental inaptitude show the disordered innervation of this "great central power."

I have used with benefit, in this condition, blisters over the abdomen, mercury, ipecac, and taraxacum combined, with enemeta of tepid water twice daily. Where it can be obtained, the warm shower bath is a valuable adjuvant. When all symptoms of inflammatory action have subsided, and the bowels are left in an atonic condition with large accumulation of gas within them, there is no medicine that can compare with the nux vomica. I prefer the alcoholic extract to any other preparation; under its use I have seen the most distressing symptoms speedily relieved.

